# Dose-dependent supplementation of *Schizochytrium* in the biofloc system modulates dual microbiomes to enhance growth and survival in Pacific white shrimp (*Litopenaeus vannamei*)

**DOI:** 10.3389/fmicb.2026.1867320

**Published:** 2026-07-22

**Authors:** Zhi Li, Xiaohan Li, Buwen Sun, Hongwei Wu, Jingyi Zhang, Xiulin Wan, Baohua Zhao, Nasi Xiao, Zhen Qi, Qingyang Li, Haiyan Liu

**Affiliations:** 1 Hebei Key Laboratory of Animal Physiology, Biochemistry and Molecular Biology, Hebei Collaborative Innovation Center for Eco-Environment, College of Life Sciences, Hebei Normal University, Shijiazhuang, China; 2 Ministry of Education Key Laboratory of Molecular and Cellular Biology, Shijiazhuang, China; 3 Genome Analysis Laboratory of the Ministry of Agriculture and Rural Affairs, Agricultural Genomics Institute at Shenzhen, Chinese Academy of Agricultural Sciences, Shenzhen, China; 4 AquaOne Tangshan Aqua-tech Co., Ltd., Tangshan, China; 5 Hebei Institute for Drug and Medical Device Control, Shijiazhuang, China

**Keywords:** biofloc microbiome, biofloc technology, gut microbiome, immune response, *Schizochytrium*

## Abstract

Integrating microalgae into biofloc system is a promising yet debated strategy in Pacific white shrimp (*Litopenaeus vannamei*) aquaculture, due to its inconsistent efficacy even for the same microalgal species. Such inconsistent performances are likely dose-dependent and the underlying microbial mechanisms remain elusive. Here, we investigated the impacts of supplementing microalga strain, *Schizochytrium* sp. ATCC 20888, at low (10^3^ cells/mL, M1) and high (10^6^ cells/mL, M2) levels compared with a clear water (CLW) system. *Schizochytrium* supplementation improved growth performance and feed efficiency and the M2 treatment further increased shrimp survival. Transcriptional profiling revealed the M2 treatment upregulated the expression of genes relevant to hepatopancreatic lipid and protein digestion (*trypsin* and *lipase*) and intestinal amino acid transportation (*peptide transporter 1*). Concurrently, M2 fortified the intestinal defense against pathogenic microbes by enhancing antimicrobial genes (*lysozyme* and *penaeidin 3a*). Redundancy analysis further supported the growth promotion was closely associated with improved lipid and a corresponding protein-sparing effect. Notably, *Schizochytrium* persisted at an extremely low abundance, whilst 16S rRNA sequencing revealed its disproportionate impact as a rare taxon on the microbiota in both biofloc and gut. High-dose supplementation enriched beneficial genera such as *Aureispira*, *Marivita*, *Neptuniibacter*, and *Phaeodactylibacter* in bioflocs, which are vital for nutrient recycling. Meanwhile, the intestinal microbiota was characterized by enrichment of the probiotic *Fusibacter* and the suppression of the opportunistic pathogen *Shewanella*. Overall, *Schizochytrium* orchestrates a dose-dependent reshaping of biofloc and gut microbiomes, effectively boosting the growth, feed efficiency, and specifically reinforcing antimicrobial activity of *L. vannamei*.

## 1 Introduction

Biofloc technology (BFT) has emerged as a revolutionary approach in aquaculture to address both productivity and sustainability (Khanjani and Sharifinia, 2020). As a microbial management system, BFT effectively transforms organic waste into a nutritious feed source while simultaneously increasing the robustness of aquatic organisms, thereby promoting an eco-friendly aquaculture paradigm (Abakari et al., 2022; Liu et al., 2025). Owing to its holistic merits and cost-effectiveness, BFT has prevailed in the aquaculture of Pacific white shrimp (*Litopenaeus vannamei*), the largest sector in global aquaculture, and urgently requires solutions to improve feed utilization, mitigate pathogen outbreaks, and prevent water quality deterioration under a superintensive density (FAO, 2024; Rego et al., 2017; Schveitzer et al., 2024; Villarreal and Juarez, 2022). However, the unstable performance of BFT has bottlenecked its further popularization. Inappropriate cultivation of biofloc will even result in the proliferation of harmful bacteria, such as Vibrio (Chen et al., 2017; Kasan et al., 2017; Tepaamorndech et al., 2020). Hence, it is of paramount importance to develop a reliable BFT system.

To optimize the biofloc system and achieve the desired outcomes, more than a dozen factors, including the carbon-to-nitrogen (C/N) ratio, carbon sources, and many others, have been extensively studied. Nevertheless, findings from relevant studies are often contradictory because of the intricate interactions among these factors and the limitations of experimental scales. For example, it is usually recommended that the input C/N ratio remain at 15:1 to increase the proportion of heterotrophic bacteria, thereby improving the conversion rate of organic waste and toxic chemicals (Avnimelech, 1999; Minaz and Kubilay, 2021; Xu et al., 2016). In other C/N ratio trials, this recommendation has been challenged, as few effects have been shown among different C/N ratios (Xu et al., 2022). This perspective is further supported by our previous meta-analysis (Li et al., 2024a). Moreover, the interplay between the C/N ratio and other factors, such as the carbon source, further exacerbates the complexity of biofloc system optimization (Li et al., 2024b; Mansour et al., 2022; Panigrahi et al., 2019). As a result, the industry has increasingly focused on the supplementation of microorganisms as the most direct and effective intervention among the numerous BFT measures (Ramasubburayan et al., 2025).

The integration of exogenous microalgae into biofloc systems represents a promising frontier offering comprehensive benefits (McCusker et al., 2023). Compared with probiotics exemplified by *Bacillus* and heterotrophic bacteria (Li et al., 2024a), microalgae demonstrate comparable, if not superior, effects on shrimp culture, particularly under conditions of limited water exchange. To date, four types of microalgae, including green algae, diatoms, yellow-green algae, and thraustochytrids, have been incorporated into shrimp biofloc systems. Silva et al. (2022) reported that *Scenedesmus obliquus* enhanced shrimp survival and growth, although its impact on water quality was minimal. Similarly, Ge et al. (2016) conducted a comprehensive comparison of *Platymonas helgolandica*, *Chlorella vulgaris*, and *Chaetoceros mulleri* in a biofloc system. All these factors contributed to shrimp health, reduced inorganic nitrogen levels, and inhibited the proliferation of vibrios. Among them, *P. helgolandica* exhibited the most pronounced growth-promoting effects, outperforming either *C. vulgaris* or *C. mulleri* consistently delivering benefits to *L. vannamei* farming.

Further studies have highlighted the synergistic potential of combining microalgae and/or probiotics. Situmorang et al. (2022) revealed that coculturing *Chaetoceros calcitrans* with *Bacillus* and nitrifying bacteria improved shrimp survival, growth, immunity, while reducing pathogen abundance. Huang et al. (2022) investigated the roles of *Nannochloropsis oculate* and *Thalassiosira pseudonana*, confirming their positive effects on water quality and the suppression of vibrios. Conversely, Pacheco-Vega et al. (2018) explored the integration of *Schizochytrium* sp. and lactic acid bacteria into shrimp biofloc systems but reported limited improvements in shrimp growth performance, health, and inorganic nitrogen levels. More concerningly, this combination was associated with an increased accumulation of vibrios, raising concerns about its threat to shrimp. Jiménez-Ordaz et al. (2021) supplemented *Navicula* sp. and/or *Grammatophora* sp. with a formula based on lactic acid bacteria and *Schizochytrium* sp. Interestingly, although a more complex formula improved shrimp survival, it paradoxically hindered growth performance. These findings highlight the challenges associated with optimizing combinations of microalgae and probiotics, which often require extensive trial-and-error experimentation. Crucially, such variable outcomes are highly likely tied to overlooked dose-dependent effects and the elusive microbial mechanisms orchestrated by specific microalgae. Consequently, greater emphasis should be placed on understanding how different supplementation levels drive microbial dynamics and subsequent shrimp performance.

In this study, we supplemented the *L. vannamei* biofloc system with the microalga *Schizochytrium* sp. ATCC 20888 to evaluate its impacts on shrimp growth performance, robustness, and water quality comprehensively. Moreover, we analyzed the microbiota in bioflocs and midguts to elucidate the role that microorganisms play in the system. *Schizochytrium* sp. ATCC 20888 is a natural strain rich in docosahexaenoic acid (DHA) and has not been previously reported in biofloc systems. Owing to its heterotrophic nature and salinity tolerance, this strain is suitable for environments characterized by low illumination and seawater conditions (Zhao et al., 2024). This study aimed to provide novel insights into the application of *Schizochytrium* sp. ATCC 20888 and reveal the underlying mechanism.

## 2 Materials and methods

### 2.1 Cultivation of microalgae

The microalga strain *Schizochytrium* sp. ATCC 20888 was generously provided by Dr. Huan Xiang from South China Sea Fisheries Research Institute, Chinese Academy of Fishery Sciences. The strain was cultivated in seawater-based medium following a standardized protocol adapted from Xu et al. (2025). To initiate cultivation, the strain was activated by inoculating it into the medium at a concentration of 5% (v/v). The culture was then incubated in the dark at a constant temperature of 25 °C with continuous shaking at 150 rpm for 48 h to promote cell activation. To ensure the stability and viability of the strain, the culture underwent 3–4 successive subcultures under identical conditions. During the cultivation process, the microalgae exhibited a characteristic color shift, transitioning from an initial white appearance to a distinct yellow pigmentation as the incubation progressed, which served as a visual indicator of successful growth and active lipid metabolism. Based on the required dosage for the biofloc system, the *Schizochytrium* cultures were harvested by centrifugation at 3,000 × *g* for 10 min. The resulting microalgal pellets were subsequently resuspended in a small volume of rearing water and evenly sprayed into tanks immediately following the water exchange process.

### 2.2 Experimental design and rearing condition

The animal study protocol was approved and monitored by the Animal Care and Use Committee of Hebei Normal University. Juvenile *L. vannamei* shrimp weighing 0.32 ± 0.018 g were acquired from AquaOne Tangshan Aqua-tech Co. (China) and transported to the laboratory at Hebei Normal University. After a 1-week acclimation period, a total of 600 shrimp were evenly distributed into 15 glass tanks (length × width × height = 60 × 30 × 40 cm, filled to 75% capacity), achieving a stocking density of approximately 740 individuals/m^3^. The tanks were randomly assigned to three groups: CLW (clear water system), M1 (supplemented with 10^3^ cells/mL every other day), and M2 (supplemented with 10^6^ cells/mL every other day). The trial was conducted for 45 days, during which the shrimp were fed three times daily (08:30, 12:30, and 16:30) with a commercial formulated feed (crude protein, 44.84%; crude fat, 7.05%; crude fiber, 3.00%; ash, 14.05%; moisture, 8.07%; Mingzhi, China) at a daily feeding rate of 3%–5% of their body weight, which was adjusted based on observed daily consumption. The C/N ratio of the feed input was approximately 9:1, following the protocol described by Avnimelech (2015).

During the trial, the rearing water was constantly aerated to guarantee that the dissolved oxygen concentration remained above 5 mg/L, and was partially exchanged at a rate of 10% per day. Salinity was maintained between 28.4‰ and 29.1‰, and the temperature was controlled within the range of 28 °C–29 °C using a 50 W automatic heating rod. The pH stabilized between 7.6 and 7.98. Considering shrimp welfare and the proliferation of photosynthetic microorganisms, the photoperiod was set to a 12-h light and 12-h dark cycle with an LED grow lamp (PAR38, Philips, Netherlands).

### 2.3 Sample collection

At the end of the trial, all shrimp were harvested, counted, and weighed to determine key performance indicators, including survival rate (SR), average daily gain (ADG), feed conversion ratio (FCR), weight gain ratio (WGR), and protein efficiency ratio (PER). These performance calculations were adjusted based on the difference between the total feed supplied and the uneaten feed. Prior to tissue collection, the sampled shrimp were anesthetized in ice water to minimize stress and ensure animal welfare. For gene expression analyses, hepatopancreas and midgut samples were collected from all experimental groups. Furthermore, midgut contents from the CLW and M2 groups were specifically collected for microbiome analysis. All collected samples were immediately snap-frozen in liquid nitrogen and stored at −80 °C. Water samples were collected from each tank on Day 40 and stored at 4 °C for the evaluation of water quality parameters. These measurements included inorganic nitrogen compounds - total ammonium nitrogen (TAN), nitrite nitrogen (NO_2_-N), and nitrate nitrogen (NO_3_-N) - as well as total suspended solids (TSS). To collect the biofloc, corresponding water samples were placed in Imhoff cones (1001-0010, Thermo Fisher Scientific, United States) for sedimentation. The bioflocs were subsequently collected from the lower tips of the cones and centrifuged at 4,000 × *g* for 5 min. The acquired biofloc samples were stored at −80 °C for microbial analyses.

### 2.4 Water condition analyses

Physical parameters, including pH, salinity, and dissolved oxygen, were monitored daily using a YSI multiparameter instrument (YSI, United States) to maintain optimal rearing conditions for the shrimp. Inorganic nitrogen compounds (TAN, NO_2_-N, and NO_3_-N) were analyzed using a Clever Chem automatic discontinuous chemical analyzer (DeChem-Tech, Germany). The TSS content was measured according to the standard methods described by AOAC (2023).

### 2.5 Gene transcription analyses by real-time quantitative PCR

Total RNA was extracted from both the hepatopancreas and midgut tissues of the shrimp using TransZol reagent (ET101–01-V2; TransGen Biotech, China) following the manufacturer’s protocol. The quality and quantity of the extracted RNA were evaluated using a NanoDrop 2000 spectrophotometer (Thermo Fisher Scientific, United States). Reverse transcription of RNA into cDNA was carried out with a cDNA Synthesis Kit (K1621; Thermo Fisher Scientific, United States), followed by RT–qPCR analysis performed on an Applied Biosystems ViiA Real-Time PCR System (Applied Biosystems, United States). To accurately assess tissue-specific responses, the target genes were divided based on their primary functions and analyzed in their respective tissues. Specifically, the transcription levels of digestive enzyme-related genes, including *amylase (amy)*, *lipase (lip)*, and *trypsin (try)*, were quantified in the hepatopancreas. Besides, genes associated with immunity, nutrient transport, and stress responses-namely *Toll-like receptor (tlr)*, *prophenoloxidase (propo)*, *sodium/glucose cotransporter 1 (sglt1)*, *peptide transporter 1 (pept1)*, *anti-lipopolysaccharide factor (alf)*, *crustin (crus)*, *lysozyme (lyz)*, *penaeidin 3a (pen3a)*, and *heat shock proteins 60 and 70 (hsp60 and hsp70)*-were evaluated in the midgut. The RT-qPCR reactions were performed using a standard SYBR Green-based protocol. The thermal cycling program consisted of an initial denaturation at 95 °C for 30 s, followed by 40 cycles of 95 °C for 5 s and 60 °C for 30 s (fluorescence collection). A final melting curve analysis was performed to confirm product specificity and rule out primer dimers. The relative expression levels of the target genes were normalized against gapdh. The specific primer sequences and data calculation procedures were conducted exactly as previously described (Li et al., 2019a,2024a).

### 2.6 Microbial analyses

Preliminary assessments indicated that the abundance of eukaryotic microorganisms, such as microalgae, in the collected samples was extremely low. Consequently, rather than employing shotgun metagenomic sequencing to profile the entire microbial community, this study focused specifically on the bacterial composition using 16S rRNA gene amplicon sequencing. Microbial genomic DNA was extracted from biofloc samples from all three groups and midgut content samples from the CLW and M2 groups using a DNeasy PowerSoil Kit (Qiagen, Germany). The DNA concentration and purity were assessed using a NanoDrop 2000 spectrophotometer (Thermo Fisher Scientific, United States). The hypervariable V3–V4 region of the bacterial 16S rRNA gene was amplified using the primer pair 338F (5′-ACTCCTACGGGAGGCAGCAG-3′) and 806R (5′-GGACTACHVGGGTWTCTAAT-3′). PCR amplification was conducted as previously described (Li et al., 2024a). The resulting PCR products were purified using a PCR Clean-Up Kit (YuHua, Shanghai, China) and quantified using a Qubit dsDNA HS Assay Kit (Q32851; Thermo Fisher Scientific, United States). Purified amplicons were pooled in equimolar concentrations, and approximately 5 μg sequencing libraries were created using a TruSeq DNA Sample Prep Kit (FC-131-1024, Illumina, United States) before being sequenced on an Illumina PE300 platform (Illumina, United States).

Raw 16S rRNA gene sequencing reads were demultiplexed, quality-filtered using fastp (v0.19.6), and merged via FLASH (v1.2.11). High-quality sequences were then denoised using the DADA2 plugin in the QIIME 2 (v2020.2) pipeline with recommended parameters, which provided single-nucleotide resolution based on error profiles within samples. The denoised sequences, referred to as amplicon sequence variants (ASVs), were rarefied to 20,000 sequences per sample to standardize sequencing depth, ensuring an average Good’s coverage of 97.90%. Taxonomic assignment of ASVs was performed using the Naive Bayes consensus taxonomy classifier implemented in QIIME 2 and the SILVA 16S rRNA database (v138).

### 2.7 Statistical analyses

Prior to statistical evaluation, all data were tested for normality and homogeneity of variances using the Shapiro-Wilk, Bartlett’s and Levene’s tests, respectively. For comparisons among multiple groups, the data were analyzed using one-way analysis of variance (ANOVA) followed by Tukey’s multiple comparisons test. For comparisons between two groups, the data were analyzed using an unpaired Student’s *t*-test. Both analyses were conducted using GraphPad Prism 9.0 (GraphPad, United States). As described by Gullian-Klanian et al. (2023), significantly different datasets identified via one-way ANOVA were further subjected to redundancy analysis (RDA) with 2,000 Monte Carlo permutations using CANOCO 5.0 (Microcomputer Power, United States). Bioinformatic analyses of biofloc and midgut microbiota (α and β diversity) were carried out and visualized using the Majorbio Cloud platform^1^, with the statistical significance of β diversity evaluated via PERMANOVA.

All results are presented as the means ± standard deviations (SD). Differences were considered statistically significant at *P* < 0.05. In the data tables, values in the same row with different superscript letters indicate significant differences (*P* < 0.05). For graphical representations, significance levels are indicated as *P < 0.05, **P < 0.01, and ***P < 0.001.

## 3 Results

### 3.1 Survival rate and growth performance

The detected SR and growth performance are presented in Table 1. Shrimp in M2 presented an approximately 15% greater SR than those in both CLW (*P* < 0.05) and M1 (*P* < 0.05). With respect to growth performance, clear dose-dependent improvements associated with *Schizochytrium* supplementation were observed across all three groups. In terms of the growth rate, both the ADG and WGR in M2 were greater than those in M1 (*P* < 0.05), which in turn were greater than those in CLW (*P* < 0.05). Similarly, the feed efficiency indicators, including FCR and PER, followed the same pattern, with M2 showing the most favorable values, followed by M1 and then CLW (*P* < 0.05 for all comparisons).

**TABLE 1:** Effects of *Schizochytrium* supplementation on the survival rate and growth performance of *L. vannamei*.

Indicator	CLW	M1	M2	*P*-value
SR, %	76.80 ± 12.80^a^	75.46 ± 5.19^a^	91.76 ± 2.96^b^	0.0018
ADG, g	0.145 ± 0.026^a^	0.224 ± 0.008^b^	0.255 ± 0.015^c^	<0.0001
FCR	2.33 ± 0.30^a^	1.76 ± 0.07^b^	1.55 ± 0.09^c^	0.0024
WGR, %	2078 ± 430^a^	3155 ± 118^b^	3591 ± 212^c^	<0.0009
PER	0.97 ± 0.13^a^	1.27 ± 0.05^b^	1.44 ± 0.09^c^	<0.0001

Different superscript letters within the same row indicate significant differences among groups (*P* < 0.05).

### 3.2 Gene expression

**FIGURE 1:**
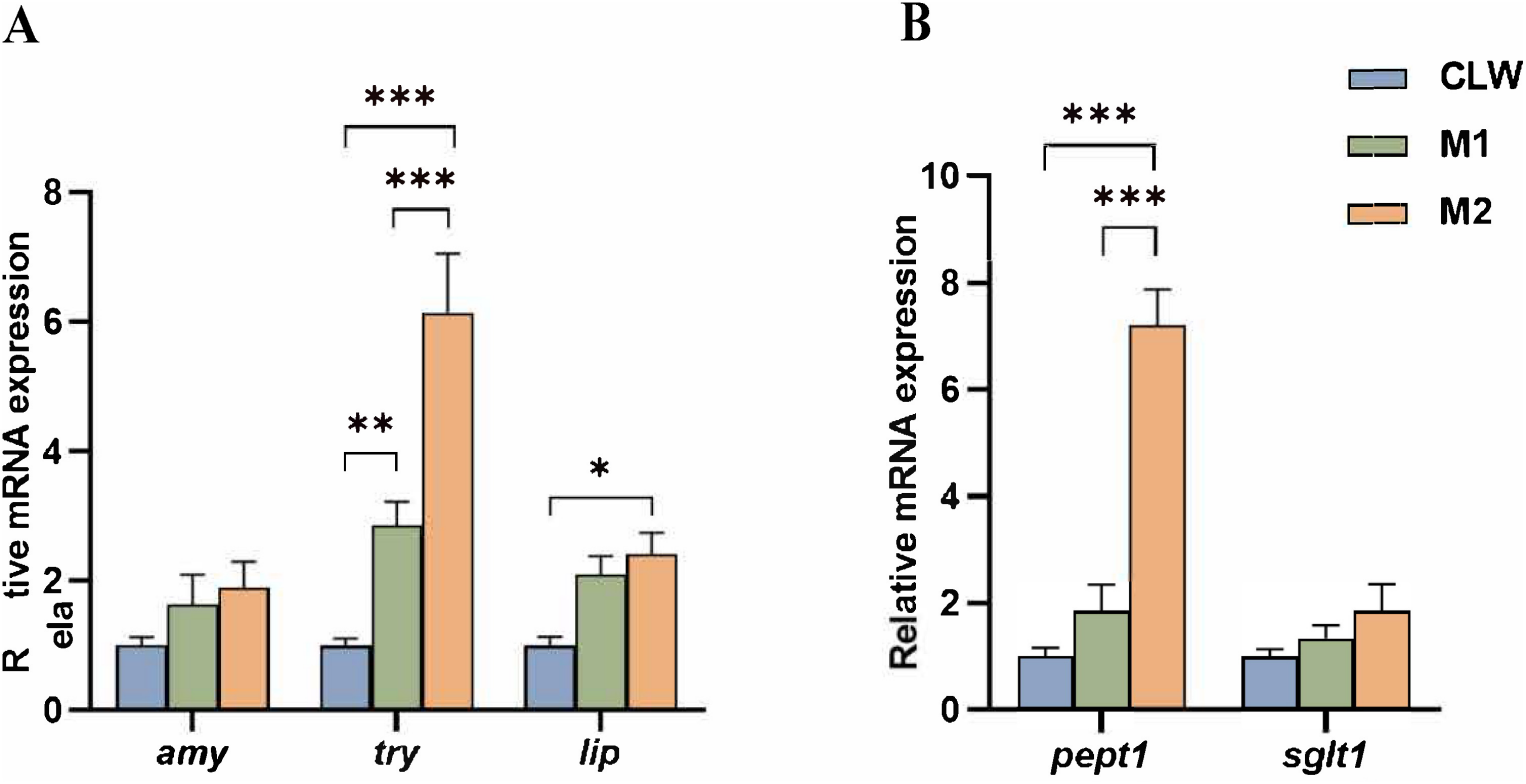
Expression profiles of genes involved in panel **(A)** hepatopancreatic nutritional digestion and **(B)** intestinal absorption. *P < 0.05, **P < 0.01, ***P < 0.001.

Figure 1 shows the expression patterns of genes relevant to shrimp growth performance, including those involved in nutritional digestion (*amy*, *try*, and *lip*) in the hepatopancreas, and absorption (*pept1* and *sglt1*) in the intestine. Supplementation with *Schizochytrium* upregulated the transcription of *try* by approximately 3-fold in M1 (*P* < 0.01) and 6-fold in M2 (*P* < 0.001). Furthermore, *try* expression in the shrimp was greater in M2 than in M1 (*P* < 0.001) (Figure 1A). In addition, *lip* expression was higher in M2 than in CLW (*P* < 0.05) (Figure 1A). With respect to genes involved in nutrient absorption (Figure 1B), M2 presented higher expression of *pept1* than both M1 and CLW (*P* < 0.001), while no difference was observed between M1 and CLW.

**FIGURE 2:**
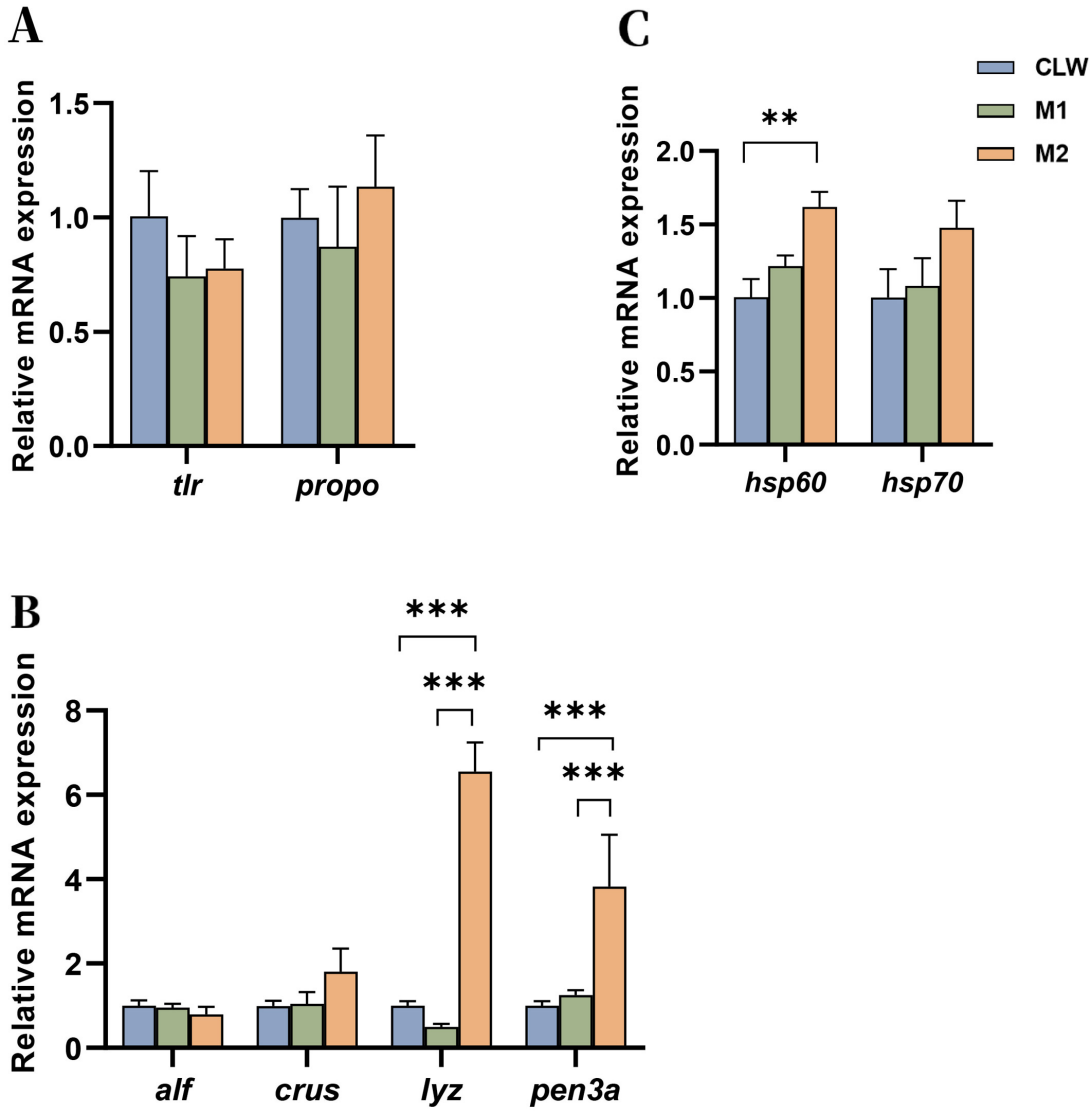
Expression profiles of genes involved in panel **(A)** pathogen recognition, **(B)** antimicrobial peptides, and **(C)** stress response. *P < 0.05, **P < 0.01, ***P < 0.001.

Figure 2 shows the expression dynamics of genes related to disease resistance, including genes related to pathogen recognition (*tlr* and *propo*), antimicrobial peptides (*alf*, *crus*, *lyz*, and *pen3a*), and the stress response (*hsp60* and *hsp70*). No changes in transcription related to pathogen recognition were detected (Figure 2A). M2 markedly amplified the expression of *lyz* by at least fivefold compared with that in CLW or M1 (*P* < 0.001). Moreover, compared with CLW and M1, M2 increased the transcription of *pen3a* nearly fourfold (*P* < 0.001) (Figure 2B). Regarding the stress response, M2 upregulated the expression of *hsp60* compared with CLW (*P* < 0.01), whereas the transcription of *hsp70* remained unaffected across all groups (Figure 2C).

### 3.3 Water quality and biofloc accumulation

**FIGURE 3:**
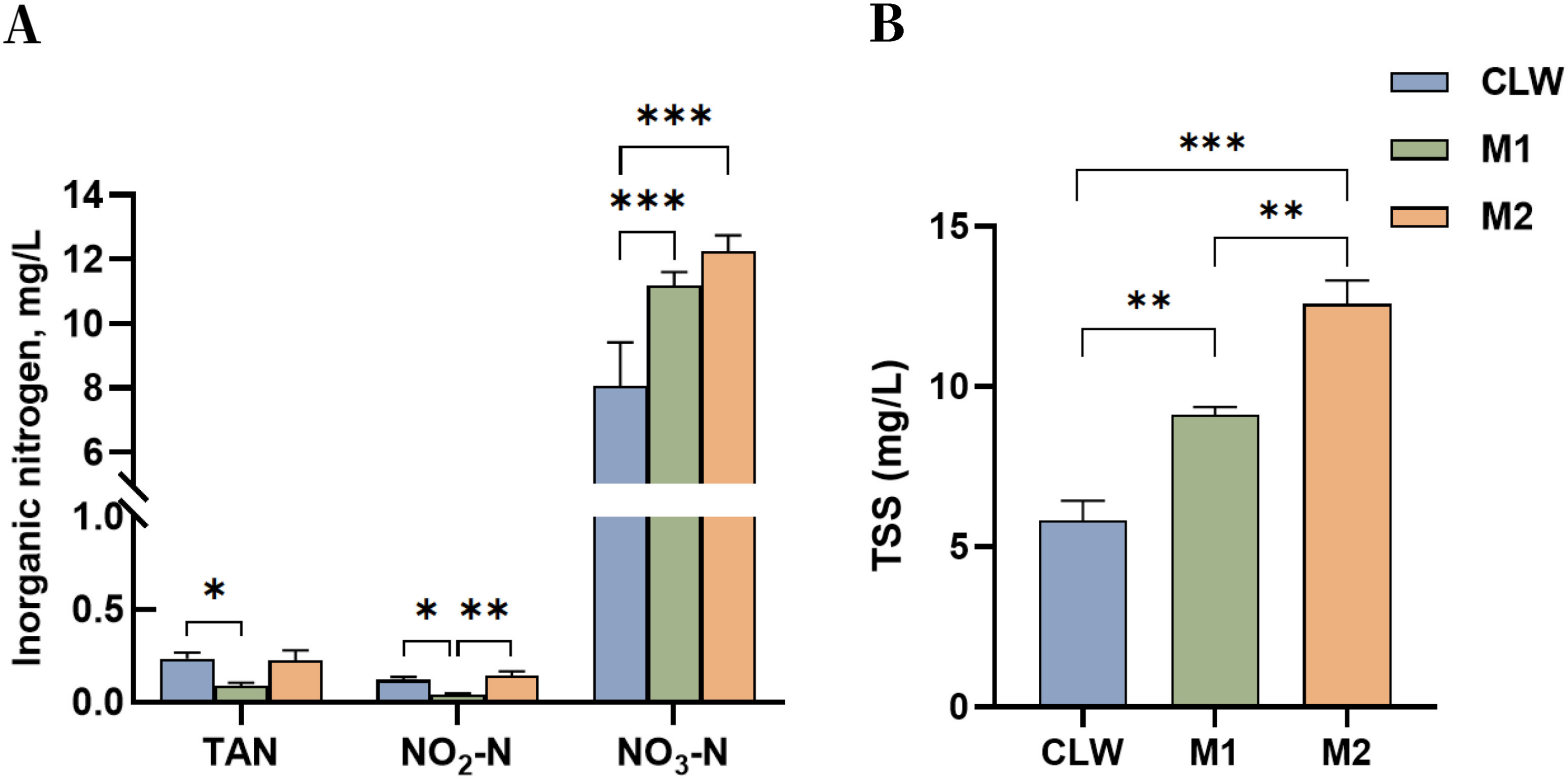
Effects of *Schizochytrium* supplementation on the content of panel **(A)** inorganic nitrogen and **(B)** total suspended solids (TSS). *P < 0.05, **P < 0.01, ***P < 0.001.

Figure 3 shows the water quality parameters (inorganic nitrogen, Figure 3A) and biofloc accumulation (TSS, Figure 3B). Compared with CLW, M1 had lower TAN and NO_2_-N contents (*P* < 0.05). The NO_2_-N content in M2 was approximately equal to that in CLW (*P* > 0.05) but higher than that in M1 (*P* < 0.01). Furthermore, both M1 and M2 presented higher NO_3_-N concentrations than CLW (*P* < 0.001) (Figure 3A). Regarding biofloc accumulation, a pronounced dose-dependent increase in TSS was evident, rising progressively from CLW to M1 (*P* < 0.01) and reaching the highest value in M2 (*P* < 0.01 compared with M1; *P* < 0.001 compared with CLW) (Figure 3B).

### 3.4 Integrative relationships among shrimp performance, gene expression, and water quality

**FIGURE 4:**
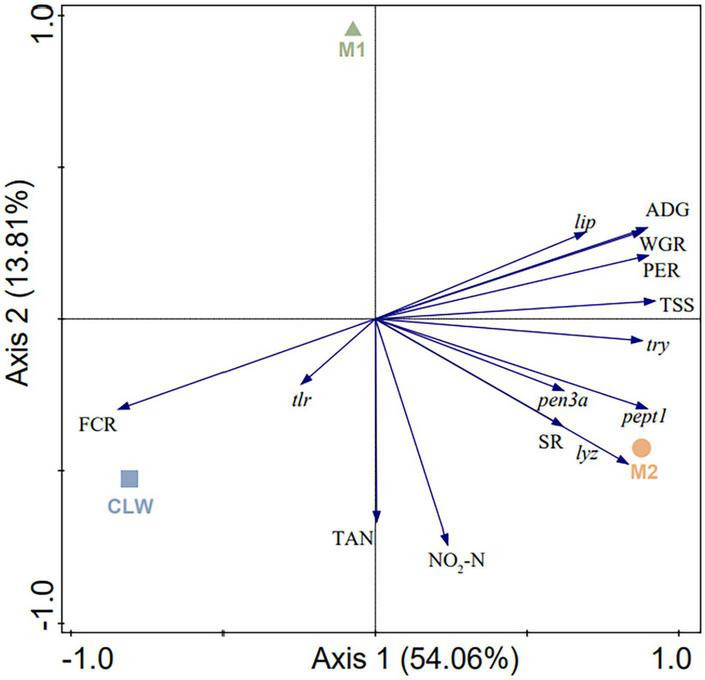
Redundancy analysis (RDA) illustrating the associations between groupings (independent variables) and indicators (predictive variables) in rearing systems.

The RDA results (Figure 4) illustrate the associations between the treatments and the response variables previously screened via one-way ANOVA (*P* < 0.05). The model explained 67.87% of the total variation (Axis 1: 54.06%; Axis 2: 13.81%; pseudo-F = 5.5). This variation was primarily driven by M2 (46.6%, pseudo-F = 11.3, *P* = 0.002) and CLW (21.3%, pseudo-F = 8.0, *P* < 0.0005). Specifically, M2 was strongly positively associated with SR and the expression of immunity-related genes (*pen3a*, *lyz*) as well as protein digestion and absorption genes (*try*, *pept1*). Furthermore, M2 was also positively correlated with TSS, PER, ADG, and WGR, but negatively correlated with FCR. Regarding variable relationships, ADG, WGR, and PER exhibited strong positive correlations with each other, as well as with *lip* and TSS. Besides, FCR was negatively correlated with these parameters. In contrast, weaker correlations were observed between growth metrics and NO_2_-N and TAN, given their nearly orthogonal vectors.

### 3.5 Biofloc diversity and compositional structure

**FIGURE 5:**
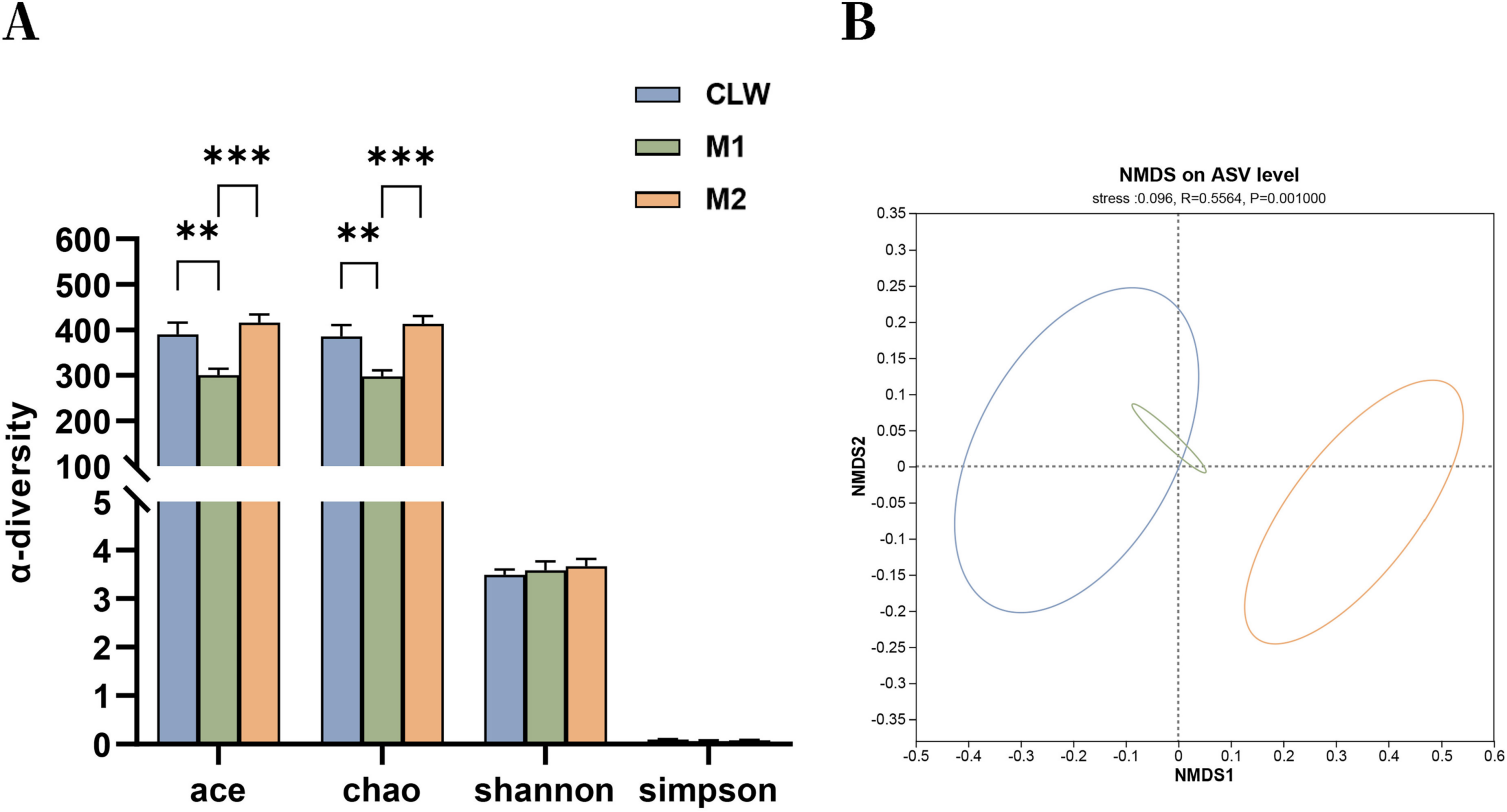
Effects of *Schizochytrium* supplementation on the biofloc microbial diversity. **(A)** α-diversity measures of Ace, Chao, Shannon, and Simpson; **(B)** β-diversity analysis represented by NMDS at the ASV level. **P < 0.01, ***P < 0.001.

Figure 5 presents the microbial diversity within the biofloc at the ASV level. Regarding α-diversity, the ACE and Chao1 indices revealed that, compared with CLW, M1 significantly reduced the microbial richness of the biofloc (*P* < 0.01). In contrast, M2 effectively mitigated this reduction (*P* < 0.001), restoring the richness to a level comparable with that of CLW (*P* > 0.05) (Figure 5A). Furthermore, the non-metric multidimensional scaling (NMDS) plot demonstrated clear clustering patterns, with a distinct separation between CLW and M2, whereas M1 considerably overlapped with CLW (stress = 0.096, *P* < 0.001) (Figure 5B).

**FIGURE 6:**
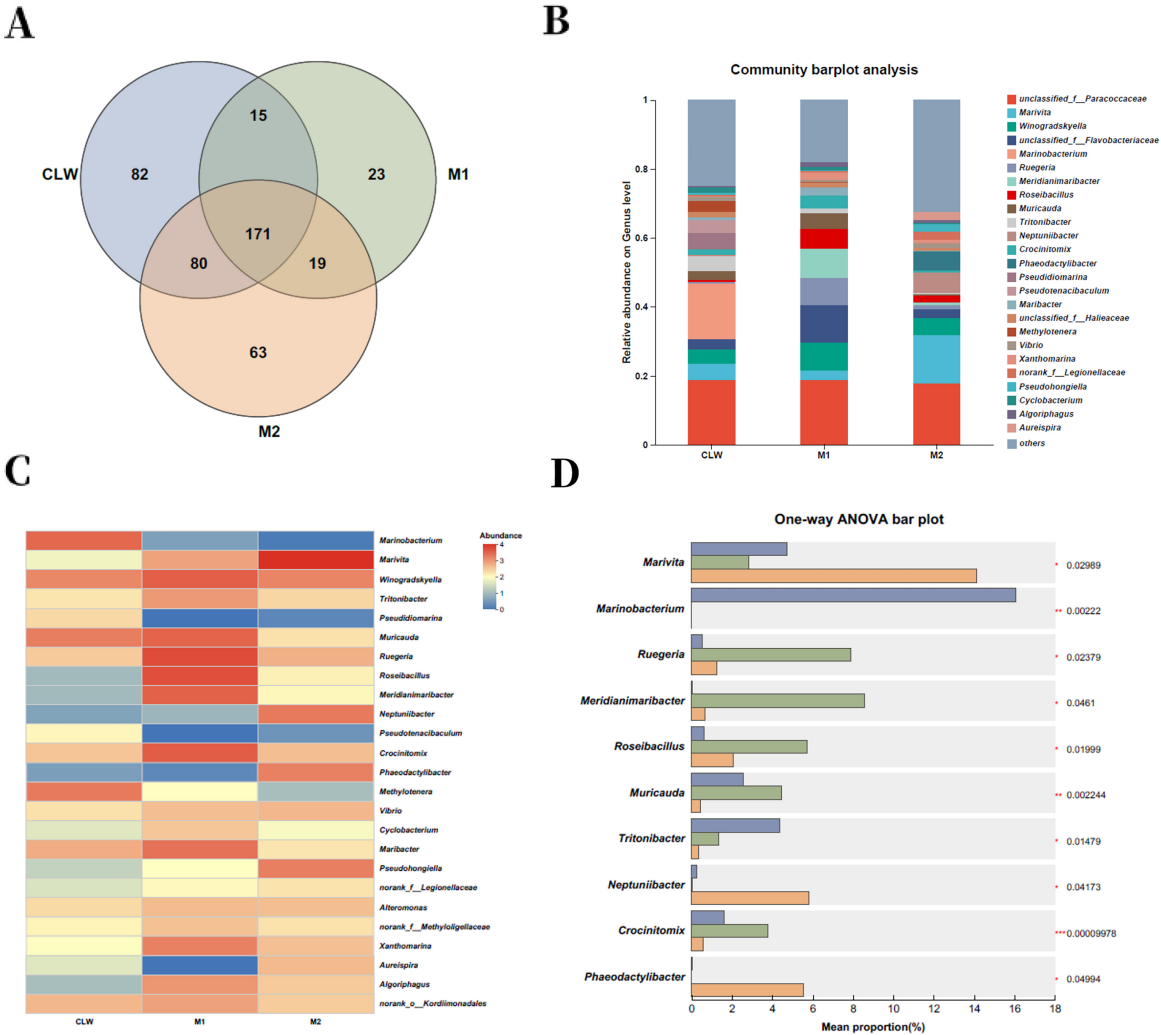
Effects of *Schizochytrium* supplementation on the biofloc microbial community structure. **(A)** Venn diagram illustrating the overlap of genera; **(B)** bar plot of the top 25 abundant genera; **(C)** heatmap of the top 25 abundant genera; **(D)** one-way ANOVA of the top 10 abundant genera. Blue, CLW; Green, M1; Orange, M2. *P < 0.05, **P < 0.01, ***P < 0.001.

Figure 6 highlights the compositional structure of the biofloc at the genus level. The Venn diagram revealed 82 unique genera in CLW, 23 in M1, and 63 in M2, with 171 genera shared across all three groups (Figure 6A). Comparative analysis of the microbial composition, as shown in the bar plot (Figure 6B) and the heatmap (Figure 6C), revealed notable trends in the relative abundances of key genera. Specifically, *Methylotenera* and *Marinobacterium* gradually declined in M1 and M2. In addition, *Aureispira*, *Neptuniibacter*, *Phaeodactylibacter*, and *Marivita* increased in abundance with the increasing addition of *Schizochytrium*. Additionally, one-way ANOVA confirmed significant differences (*P* < 0.05) in the relative abundances of several dominant genera, notably *Marivita* and *Neptuniibacter* (Figure 6D).

### 3.6 Gut microbiota diversity and compositional structure

**FIGURE 7:**
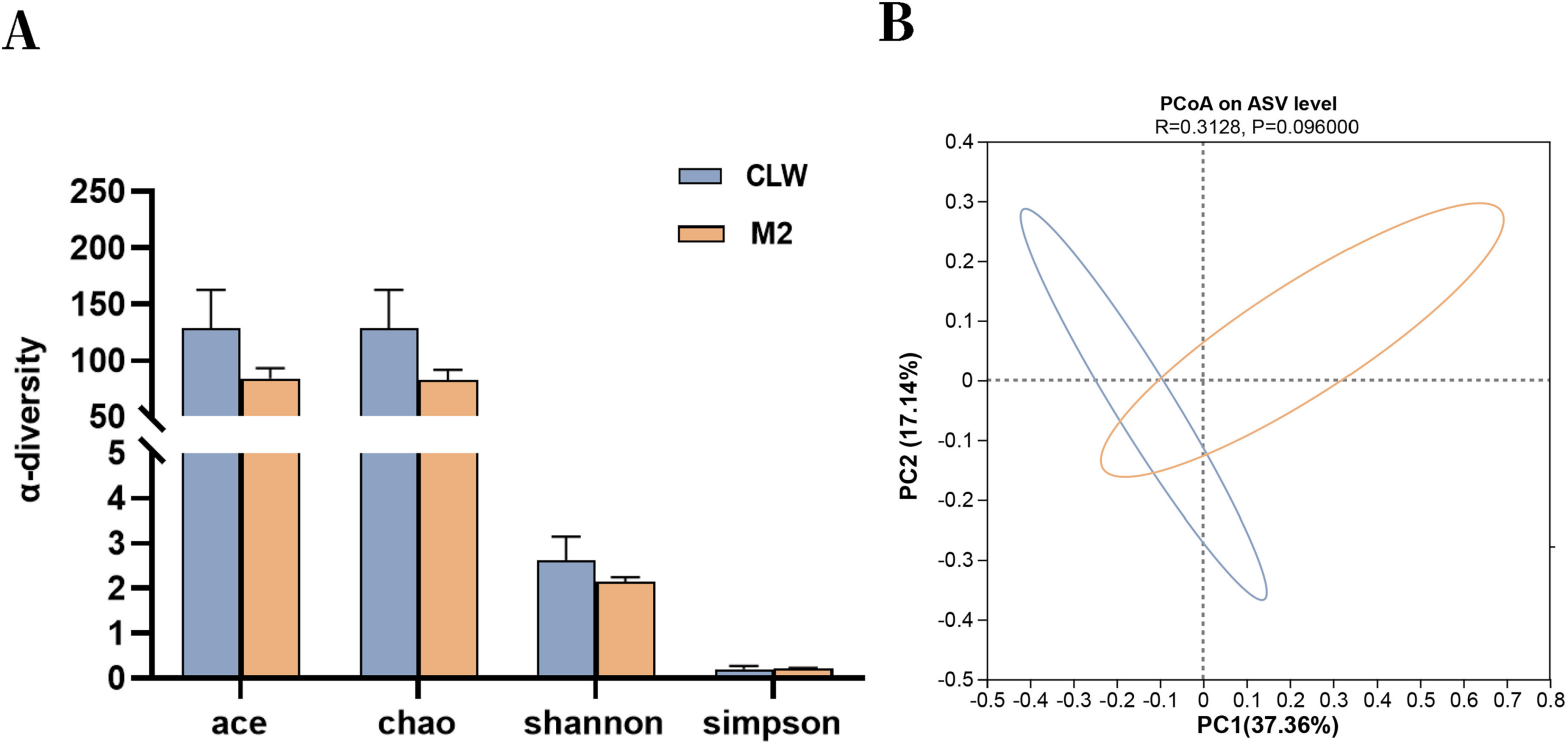
Effects of *Schizochytrium* supplementation on the intestinal microbial diversity. **(A)** α-diversity measures of Ace, Chao, Shannon, and Simpson; **(B)** β-diversity analysis represented by PCoA at the ASV level.

Figure 7 depicts the gut microbiota diversity at the ASV level between CLW and M2. The introduction of *Schizochytrium* did not affect microbial diversity, as no variations were detected in the α-diversity indices (*P* > 0.05) (Figure 7A). Nevertheless, the addition of *Schizochytrium* appeared to impact the compositional structure of the shrimp gut microbiota, as the PCoA plot revealed a divergence between CLW and M2 (*P* < 0.01) (Figure 7B).

**FIGURE 8:**
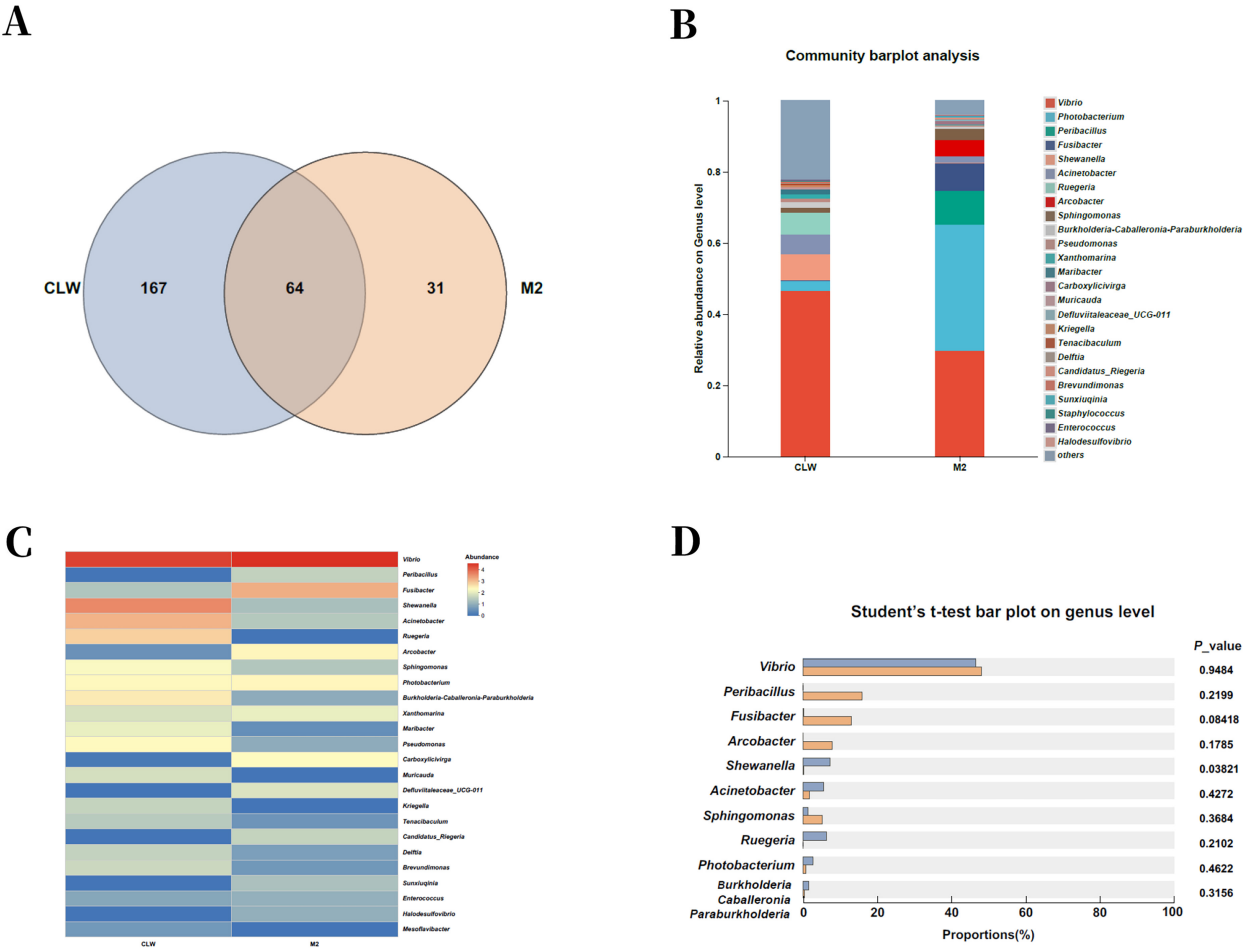
Effects of *Schizochytrium* supplementation on the intestinal microbial community. **(A)** Venn diagram illustrating the overlap of genera; **(B)** bar plot of the top 25 abundant genera; **(C)** heatmap of the top 25 abundant genera; **(D)** Student’s *t*-test of the top 10 abundant genera. Blue, CLW; Orange, M2.

Figure 8 delves deeper into the differentially abundant microorganisms between CLW and M2. The Venn diagram shows that CLW and M2 contained 167 and 31 unique ASVs, respectively, whereas 64 ASVs were shared between the two groups (Figure 8A). Both the bar plot (Figure 8B) and the heatmap (Figure 8C) revealed a marked reduction in the relative abundance of *Shewanella*, alongside an increase in *Fusibacter*. This pattern was further corroborated by Student’s *t*-test, which indicated a decrease in *Shewanella* (*P* < 0.05) and an upward trend for *Fusibacter* (*P* < 0.1) (Figure 8D).

## 4 Discussion

Addressing the inconsistent efficacy of microalgae in BFT systems, our study investigated the dose-dependent effects of a BFT system supplemented with the microalga *Schizochytrium* sp. ATCC 20888 on the growth performance and health condition of *L. vannamei* as well as the water quality. Additionally, we analyzed the microbial composition within both bioflocs and shrimp intestinal tracts to elucidate how this microalga orchestrates the causal relationships between microorganisms and the overall outcomes of the system. As a critical premise, the stocking density far exceeded the criteria for superintensive farming of *L. vannamei*, ensuring the practical applicability and reliability for industrialized shrimp farming (Villarreal and Juarez, 2022). Our findings reveal a clear divergence in system outcomes based on the supplementation level. While a lower load of *Schizochytrium* (M1: 10^3^ cells/mL, every other day) improved water quality but yielded similar production efficiency as the CLW treatment, a high level of *Schizochytrium* supplementation (M2: 10^6^ cells/mL, every other day) manipulated a biofloc system with holistic benefits, including improved shrimp growth performance and survival, together with maintenance of comparable levels of TAN and NO_2_-N. Given the relatively high SR and tolerable content of inorganic nitrogen in shrimp (Lin and Chen, 2003; Pimentel et al., 2025; Prates et al., 2024), adopting a relatively high dose of *Schizochytrium* is a highly favorable trade-off for maximizing overall production and warrants further investigation.

Our study further revealed that the ability of high-dose *Schizochytrium* supplementation to promote shrimp growth relies not only on conventional nitrogen recycling but also on a protein-sparing effect driven by lipid utilization. As reflected by the RDA and RT-qPCR results, the growth-promoting effects observed in the current biofloc system were closely associated with the effective utilization of lipids (*lip*) and biofloc accumulation (TSS). Interestingly, the hepatopancreatic enzyme involved in protein digestion (*try*) and the intestinal transporter responsible for amino acid uptake (*pept1*) appeared to play secondary roles in shrimp growth, as they exhibited weaker correlations with growth metrics than *lip* did. Moreover, unaffected or slightly deteriorated water quality (such as NO_2_-N and TAN) had a minimal impact on shrimp growth performance given their nearly orthogonal vectors in the RDA plot. Conventionally, the growth-enhancing effects of biofloc systems are attributed to the reutilization of ingestible nitrogen substances, improved nutrient utilization efficiency, and elevated water quality (Khanjani and Alizadeh, 2023). Instead, our findings support an alternative mechanism where an increased availability of fatty acids derived from the modulated biofloc can slow gastric emptying. This physiological shift prolongs the interaction between nutrients and both digestive and transport enzymes, ultimately increasing amino acid digestibility, thereby enhancing overall growth performance (Cervantes-Pahm and Stein, 2008; Bromley, 1994; Li and Sauer, 1994). As a result, a greater proportion of amino acids is directed toward meat production, thereby contributing to improved weight gain and feed efficiency (Li et al., 2019b; Luan et al., 2023). Hence, the inclusion of *Schizochytrium* in the shrimp biofloc system enhances growth performance primarily through increased lipid availability and subsequent energy reallocation, rather than merely relying on improved protein supply or water quality.

Simultaneously *Schizochytrium* supplementating in the shrimp biofloc system exerts immunostimulatory effects in a highly efficient manner (El-Sayed, 2021). Biofloc system is widely recognized for its capacity to ameliorate symptoms of acute hepatopancreatic necrosis disease and white spot syndrome virus, which remain the most severe threats to the shrimp industry (Ekasari et al., 2014; Hostins et al., 2019; Kumar et al., 2020). Generally, this system fosters a holistic improvement in disease resistance by enhancing antioxidant activity (Liu et al., 2017), pathogen recognition (Li et al., 2024a), and antimicrobial capacity (Gustilatov et al., 2022). These benefits to shrimp robustness are primarily based on elevated humoral activity and the expression of key immune genes (Tepaamorndech et al., 2020; Xu and Pan, 2013). Crucially, the transcriptional dynamics observed in our study indicate that shrimp prioritize the intensification of direct antimicrobial activity (*lyz* and *pen3a*) over broad pathogen recognition (*tlr* and *propo*), thereby fortifying their frontline defense against infections (Ghosh et al., 2024; Pilotto et al., 2020). This sophisticated and targeted defense strategy minimizes physiological costs and conserves more energy and nutrients for somatic development, partially explaining the improved growth of shrimp in the supplemented system (Bird, 2019).

Acting much like an orchestra conductor, the supplemental microalga *Schizochytrium* shapes microbial dynamics to enhance the overall biofloc system (Chakraborty et al., 2025). While these systems are inherently driven by microbes and are conventionally thought to be easily manipulated by dominant exogenous species (Raza et al., 2024), our current findings and recent reports contradict this assumption. Specifically, exogenous microbes rarely achieve absolute dominance within either the biofloc or the intestinal microbiome (Li et al., 2024a). Consequently, *Schizochytrium* likely operates as a rare taxon, exerting a disproportionate influence to remodel the entire community architecture despite its extremely low relative abundance (Chakraborty et al., 2025; Shade and Gilbert, 2015). Driven by this hypothesis, our analysis of the biofloc microbial variations revealed distinct bacterial shifts corresponding to the algal supplementation dosage. Notably, prominent genera including *Aureispira*, *Marivita*, *Neptuniibacter*, and *Phaeodactylibacter* exhibited an enrichment proportional to the algal input. These specific bacteria actively participate in nutrient recycling, immune stimulation, and water purification. Beyond providing beneficial metabolites, these enriched populations play crucial roles in modulating the host gut microbiome. Conversely, genera such as *Methylotenera* and *Marinobacterium* faced competitive inhibition, likely due to a lack of accessible nutrients in the altered ecological niche (Kamira et al., 2018; Liu X. et al., 2024; Satanwat et al., 2020).

Among these enriched taxa, *Aureispira* emerges as a particularly noteworthy bacterium due to its unique trophic strategies. Although rarely reported in previous biofloc research, this special nutritional mode endows *Aureispira* with the ability to access nutrients effectively within heterogeneous aquatic environments. Such adaptability is a hallmark of biofloc networks and makes *Aureispira* crucial for structuring the broader microbial population (Lien et al., 2024). When encountering pathogens such as *Vibrio parahaemolyticus*, *Aureispira* actively eliminates the threat and captures the released cellular materials (Yeoh et al., 2021). Crucially, this genus is highly proficient at synthesizing arachidonic acid (ARA) and diterpenoids (Furusawa et al., 2015). The microbial production of ARA perfectly corroborates our earlier hypothesis regarding the indirect supply of fatty acids, which subsequently enhances growth performance, elevates immunity, and modulates the gut microbial composition of *L. vannamei* (Duan et al., 2022; Qi et al., 2022). Moreover, the secreted diterpenoids possess various pharmacological properties, potentially improving shrimp health and inhibiting the proliferation of competitors to *Aureispira* (Liu X. J. et al., 2024; Shi et al., 2025).

Alongside *Aureispira*, *Marivita* typically thrives in such conditions and actively participates in aerobic anoxygenic photosynthesis, organic degradation, and nitrogen cycling (Huang et al., 2024; Zhou et al., 2023). It also secretes digestive enzymes that facilitate the degradation of complex macromolecules (Zheng et al., 2019). *Neptuniibacter* serves as another probiotic resident in biofloc that drives nitrogen and sulfur recycling (Aguilar-Rendón et al., 2022; Krejčík et al., 2008). Additionally, *Phaeodactylibacter* functions as an aerobic heterotrophic bacterium with efficient nitrifying and denitrifying capabilities (Chen et al., 2023; Lu et al., 2024). It also accelerates biofloc formation and coexists synergistically with microalgae (Chen et al., 2014; Dong et al., 2022). This documented ability directly explains the pronounced increase in the TSS content observed in our high-dose treatment. Ultimately, *Schizochytrium* species establish a highly optimized biofloc system distinguished by efficient nutrient recycling, robust biofloc formation, and abundant supplies of polyunsaturated fatty acids and bioactive compounds. Although the ecological contributions of these enriched bacteria to the biofloc system have been somewhat clarified, the precise molecular dialogues through which microalgae facilitate their enrichment still require in-depth investigation (Iqhrammullah et al., 2024).

Following *Schizochytrium* supplementation in the biofloc system, the gut microbiome of the shrimp undergoes beneficial modulation, thereby enhancing host health and facilitating adaptation to nutritional changes. Interestingly, the overall structural stability suggests that the core intestinal microecology possesses strong resilience against environmental changes (Tzeng et al., 2015). Nevertheless, targeted compositional shifts involving specific taxa such as *Fusibacter* and *Shewanella* underscore the profound systemic impact of microalga. In the shrimp midgut, *Fusibacter* contributes to the degradation of complex compounds such as cellulose and proteins, thereby increasing physiological resilience and promoting growth (Liang et al., 2022). Crucially, the intestinal enrichment of *Fusibacter* actively advances lipid metabolism (Wang et al., 2024). Serving as a physiological adaptation to an increased influx of ingested lipids, this finding provides compelling *in vivo* evidence for the accumulation of fatty acids within the system, further validating our earlier hypothesis (Li et al., 2019a). In contrast, the notable suppression of *Shewanella*, a well-known opportunistic pathogen associated with early mortality syndrome, mitigates the risk of disease outbreaks (Prachumwat et al., 2020).

## 5 Conclusion

In conclusion, our study demonstrates that a biofloc system orchestrated by an elevated abundance of *Schizochytrium* (10^6^ cells/mL) increases the growth rate and feed efficiency of *L. vannamei*. Operating in a highly efficient manner, the system specifically strengthens direct antimicrobial activity, thereby improving overall shrimp survival. These macroscopic benefits are fundamentally driven by the targeted modulation of microbial compositions in both the aquatic environment and the host midgut. Acting as a rare taxon, *Schizochytrium* effectively leverages the biofloc community, enriching key genera such as *Aureispira*, *Marivita*, *Neptuniibacter*, and *Phaeodactylibacter*. Crucially, *Aureispira* supplies arachidonic acid to the system, driving a lipid-based protein-sparing effect. This nutritional shift is further validated *in vivo* by the intestinal enrichment of the lipid-metabolizing probiotic *Fusibacter* and the concurrent suppression of the pathogen *Shewanella*. Future research should focus on deciphering the precise molecular dialogues through which microalgae facilitate the enrichment of these specific beneficial microbes across different ecological niches.

## Data Availability

The raw data generated in this study can be found in the Genome Sequence Archive (GSA) (https://ngdc.cncb.ac.cn/gsa), accession number CRA042989.

## Footnotes

1
https://cloud.majorbio.com
